# P-1305. Identifying Animal Exposures from Clinical Documents Using Large Language Models

**DOI:** 10.1093/ofid/ofae631.1486

**Published:** 2025-01-29

**Authors:** Kelly Peterson, Joann A Vuong, Christian D Dalton, Vanessa W Stevens, Makoto M Jones

**Affiliations:** Dept of Veterans Affairs; University of Utah, Salt Lake City, Utah; Veterans Affairs, Salt Lake City Health Care System; University of Utah, Division of Epidemiology, Salt Lake City, Utah; Veterans Affairs, Salt Lake City Health Care System; University of Utah, Division of Epidemiology, Salt Lake City, Utah; University of Utah, Salt Lake City, Utah; IDEAS Center of Innovation, VA Salt Lake City Health Care System, Salt Lake City, Utah

## Abstract

**Background:**

Information about animal exposure is crucial to understanding zoonotic infectious diseases; however, it is typically unavailable in structured clinical data. We evaluated the automated extraction of animal exposure mentions from clinical notes and summarized initial observations.

**Figure 1.**
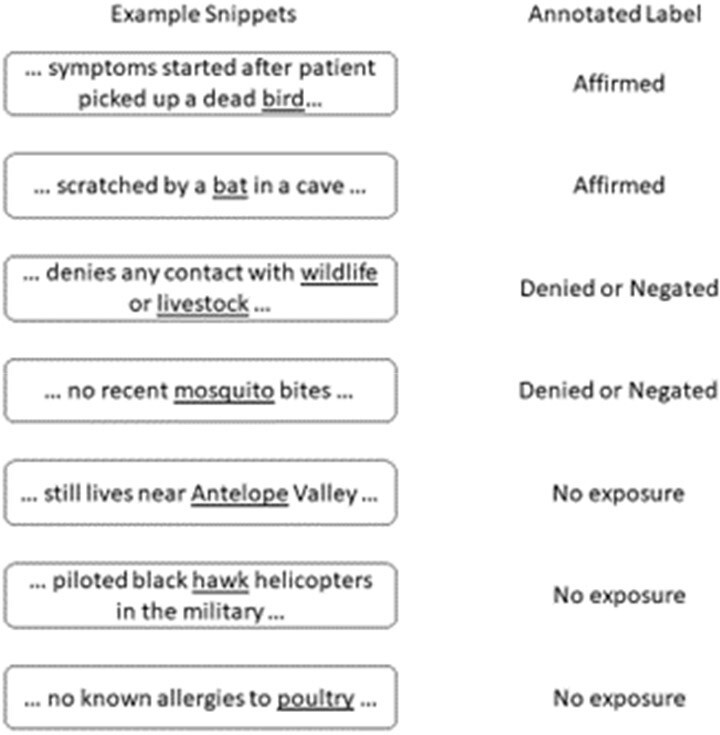
Example snippets and labels assigned during annotation. While some mentions are Affirmed exposure, others be Denied or Negated. Other keywords may not be related to animals or exposure at all.

**Methods:**

Clinical notes from the Department of Veterans Affairs (VA) data were extracted if associated with indicators for diseases which included CDC Nationally Notifiable Diseases (NND) as well as early cases of COVID-19 and mpox (i.e., positive labs, chart review, or diagnosis codes). These were then processed into text snippets containing animal keywords (e.g., cats, cattle, birds, etc.) and given to annotators who then assigned a category of: affirmed, denied/negated, or no exposure, including pets and farm animals. To augment the size of our training set, large language models (LLM) were also used to generate snippets for each category. A machine learning classification model was trained using a LLM and few-shot learning. After validation, this model was used to infer animal exposure mentions among infectious disease cases which were not part of annotation to examine the distribution of animals mentioned.

**Figure 2.**
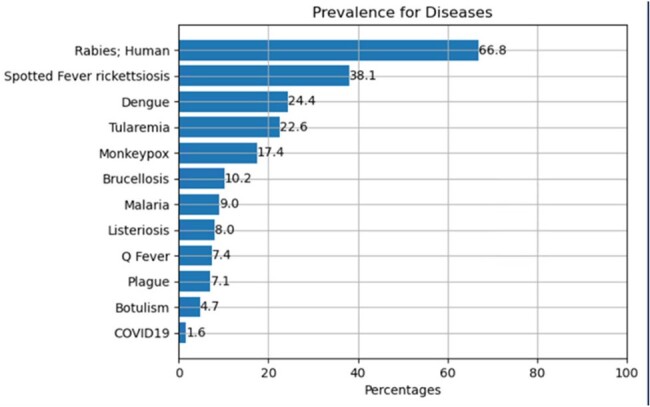
Prevalence of Affirmed Animal Exposures by disease category among documents which had at least one animal keyword so that the model could perform a classification.

**Results:**

The model’s accuracy on the held out test set of 115 (40%) text instances was 90% PPV and 91% sensitivity. Example snippets and annotations are shown in Figure 1. Following validation, the model was applied to 82,937 documents related to a selection of disease categories. The distribution of classified exposures among these documents was 2.7% affirmed, 0.1% denied/negated, and 97.2% no exposure. Figure 2 shows that prevalence of affirmed exposure varied by disease where non-zoonotic diseases such as COVID-19 were relatively lower. Some top animals terms in affirmed exposures are shown in Figure 3. Figure 4 is a distribution of top terms affirmed in tularemia cases.

**Figure 3.**
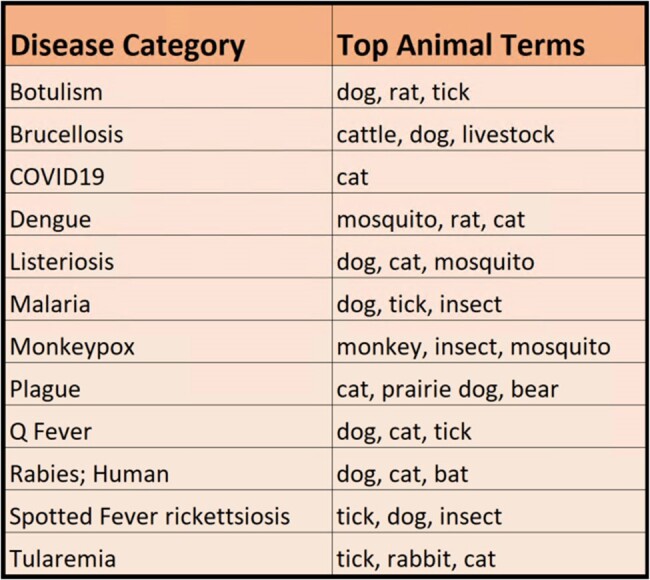
Top three animal terms associated with affirmed exposure according to inferences in the proposed model for each disease. A mention in text is simply an exposure and does not imply that the animal caused an infection. Since pets can be documented in notes along with other social history information, “dog” and “cat” may occur in any clinical note regardless of etiology.

**Conclusion:**

Automated extraction of animal exposure is feasible with small, manually generated training sets and acceptable accuracy. Our approach summarized exposures documented among several categories of infectious disease. While this work was performed retrospectively, it has been used to enhance case-finding for the biosurveillance of zoonotic diseases.

**Figure 4.**
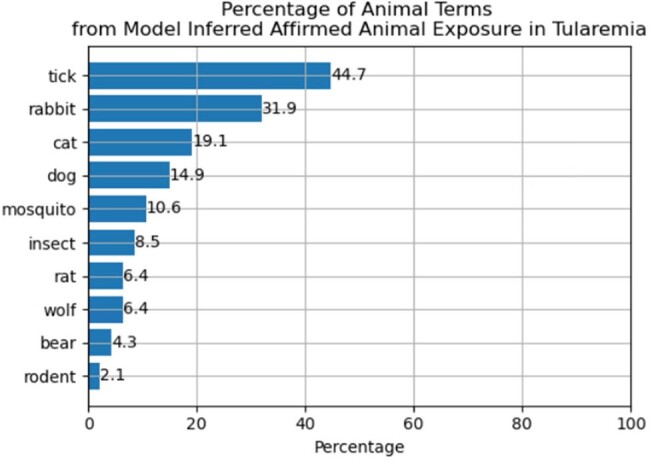
Percentages of animal terms associated with affirmed exposure according to inferences in the proposed model among tularemia cases. A mention in text is simply an exposure and does not imply that the animal caused an infection.

**Disclosures:**

**All Authors**: No reported disclosures

